# Current News and Opinion

**Published:** 1886-08

**Authors:** 


### Current News and Opinion.

### A CASE IN PRACTICE.

The following case in practice brought to my notice by A. Rosenthal, Liege, appears to me to be of sufficient interest to merit a place in the INDEPENDENT PRACTITIONER: Madame X, age thirty, suffered from a sarcoma of the lower jaw, which necessitated the resection of the ramus and a considerable portion of the body of the jaw of the right side. The second and third molars were lost by the resection. As is usual in such cases, the remaining portion of the lower jaw was so drawn to one side (in this case of course the left), as to completely destroy the articulation of the teeth, and to render every attempt at mastication fruitless. The articulation was perfectly restored by an apparatus designed by Mlle. Rosenthal, and inserted by her father.

The apparatus consisted of a small gold piece attached to the second molar and second bicuspid above (the first molar is wanting), and a similar piece attached to the bicuspids below. The lower piece carries a curved tube about two centimetres long, the upper an arm which passes into this tube. The arm is sufficiently long to prevent its being drawn quite out of the tube when the mouth is opened wide, and sufficiently strong to withstand the action of the muscles of the opposite side. This very simple apparatus completely restored the articulation and power of mastication. It has now been in use for three months. W. D. M.

Berlin, June 6th.

### INFLAMMATION IN EGGS.

There is a condition of the egg, very little known, which considerably impairs its sanitary value as an article of food. Soon after it became the practice to transport eggs in large quantities and to long distance by railway trains, it was found on their arrival that adhesion had taken place between the membranes of the yolk and those of the shell, so that the yolk could not be turned out of the shell unbroken. On examination by experienced pathologists this was found to be the result of true inflammation; the material of the adhesion was found to be precisely the same as that of the plastic exudation in inflammation of the lungs or bowels. It will at first seem absurd to speak of inflammation in such an unformed mass as an egg; but this arises from our forgetting that, structureless and unorganized as it seems, the egg, even when fresh laid, is a living being capable of disease from external causes The cause of this inflammation is undoubtedly the shaking and friction from the motion of the cars, and it cannot but render the egg more or less unhealthy, as the products of inflammation can never be as salutary in food as those of healthy growth.—*Bulletin of the Tennessee Board of Health.*

### CORRIGENDUM.

In the article entitled "Teeth with Exposed Pulps," page 355, line 12, in the July No. of the PRACTITIONER, please read "or a foramen enlarged from *disease;*" for, "a foramen enlarged from *disuse.*"

B. MERRILL HOPKINSON, D. D. S , M. D , Baltimore, Md.

### AMERICAN DENTISTS IN GERMANY.

The excellence of American dentistry is nothing new to Europeans, but it is not often that such tangible testimony is borne to it as is involved in the establishment of a German journal as the express exponent of the thought and interests of the German dentists educated in America. Such is the function of the "Journal fur Zahnheilkunde, Vereins-Organ der Deutschen Vereinigung in Amerkia graduister Doctoren der Zahnheilkunde," the first number of which has just reached us. The new journal, which is to be a quarterly at first, with the prospect of more frequent publication in the future, is edited by Dr. Erich Richter, and published in Breslau. The initial issue is handsome in appearance, and is filled with excellent matter. We have no doubt of its prosperity, and we wish it the fullest measure of success, quite as much for its own merit as out of gratification at the implied honor to our country.—*New York Medical Journal.*

### MISSOURI STATE DENTAL ASSOCIATION.

The following members were elected to serve as officers for the ensuing year:

*President*—Wm. N Conrad, St. Louis.

*First Vice-President*—R. R. Vaughn, Marshall.

*Second Vice-President*—T. M. Nicholson, Fayette.

*Recording Secretary*—John G. Harper, St Louis.

*Corresponding Secretary*—Geo. L. Shepard, Sedalia.

*Treasurer*—Jas. A Price, Weston

JOHN G. HARPER, Recording Secretary,
516 Walnut St , St. Louis, Mo.

### NATIONAL ASSOCIATION OF DENTAL FACULTIES.

By request of the Executive Committee, the time for holding the Annual Meeting of the National Association of Dental Faculties has been changed from August 4th, to Monday, August 2d, 2 P. M , at Niagara Falls.

H. A. SMITH, Secretary,
Cincinnati, O.

THE WEEKLY MEDICAL REVIEW, we take it, is not much in favor of home rule. Quoting a paragraph from last month's INDEPENDENT PRACTITIONER, concerning the expense of municipal government in the different great cities of the world, in which it was shown that it cost $36.95 *per capita* in New York, as against $7.40 in London, $7.35 in Berlin, and $5.40 in Paris, it intimates that London is governed by Englishmen, Berlin by Germans, and Paris by Frenchmen, while New York is ruled by the Irish. That *is* rather a reflection on "Home Rule."

MAJOR R. E. STEWART, L. D. S., of Liverpool, England, died in that city, May 19th. He was a liberal supporter of dental movements, and held the Chair of Mechanical Dentistry in the Liverpool School of Medicine. A number of the delegates to the Congress of 1881 will remember with pleasure the convivial Major.

The deaths from anæsthesia, during the past year in England, have been reported in *The British Medical Journal* Twelve deaths occurred from chloroform, eight of the operations during which the agent was used having been severe. From ether but three died, which is remarkable considering that it is used very largely in England. No death has been recorded from the bichloride of methylene, nor any from mixtures of chloroform and ether. This, too, is noteworthy, the A. C. E. mixture—alcohol, chloroform and ether—being a favorite one with English surgeons. As there is nothing in either that is antidotal to the other, it might be anticipated that the mixture would combine the dangers of each. This, however, does not seem to be the case, the immunity being probably due to the dilution of the drugs by the alcohol.

---

Dr W. H. Waite, of Liverpool, England, says *The Journal of the British Dental Association,* is compelled to retire from practice because of a threatened loss of vision. This is a matter of regret to every dentist who loves his profession, but to those who know the man the knowledge will come as a personal affliction. Dr. Waite has visited this country more than once, the last time being only two years ago, and there were few foreign dentists who had so many personal friends and admirers in America. His mental qualifications, his moral worth and his genial manner, have gained him the respect and regard of all whom he has met in America, and they will unite in the wish that the present dark anticipations concerning his vision may not be realized.

---

In this number will be found a continuation of Dr. Harlan's "Observations on Foreign Dental Schools," the first part of which was published in the May number of this journal. *The Journal of the British Dental Association* speaks of the first article as follows : "Dr. Harlan has published a very chatty and agreeably written account of his recent experience in Europe, in the Independent Practitioner. The article is to be followed up by another on the same subject. It is well worth reading, and has the merit of being just and impartial in its criticisms. The interest of a review of our own doings by a friendly visitor warrants us in extracting the article *in extenso.*"

---

Sir Henry Thompson believes that artificial teeth are an evil to those advanced in years, as they enable such persons to masticate flesh. When the teeth fail naturally, he says, it is nature's design that the individual should subsist on vegetable diet.

What a crank. Everyone knows that flesh is more easily digested than vegetables. Besides, the latter need mastication and insalivation more than the former, if possible. It is certain that Sir Henry Thompson is not an old and toothless man, and that when he becomes so he will let no time be wasted in procuring a set of false teeth.

---

On February 16th was sold by auction, at Kensington, the historical house once occupied by John Hunter. Among the articles advertised for sale was "the old historical copper coving and fittings," used for the purpose of boiling the remains of the Irish giant, O'Brian.

The President has vetoed the bill legalizing dissection in the District of Columbia, on the ground that it failed to provide sufficient safeguards against the delivery of bodies to unauthorized persons.

The President uses the veto hatchet like—a lawyer. Medical men are liable to suits from lawyers if they are not thoroughly familiar with human anatomy, and yet they would remove the only possible source of practical anatomical knowledge.

---

Those who desire to find a hotel in New York which will give them all the comforts of a home, will do well to visit "The Westminster," at Sixteenth Street and Irving Place, only a block from Union Square. Its table is unrivalled. A party of six gentlemen lately dined there with a guest of the house, the dinner being served from the regular bill of fare, and it was with difficulty that the host of the occasion could convince his friends that it was not a formal affair, specially ordered for the occasion.

---

Archives of Pediatrics is an excellent journal from a medical point of view, and unexceptionable from a literary standpoint, but morally it is worthy of rebuke. There is a wide difference between a plain and faithful medical description of any given case and unnecessary vulgarity—between the use of essential anatomical terms and the descent to disgusting obscenity. The author of the leading article in the June number violates common decency.

---

If there is any meanness in a man or woman, it is certain to show itself in traveling. At home the usage of good breeding demands common politeness, but on a journey selfishness exhibits itself, for then it is everyone for himself. I would never marry a woman till I had traveled with her. I would prefer the wedding trip before marriage. It might be a voyage of valuable discoveries. —*Lancet and Clinic.*

---

It will bring pain to many hearts to know that Bradford C., only son of Dr. F. E. Howard, of Buffalo, was drowned at Geneseo, July 9th. Young Howard was a very bright and promising boy of fourteen years. The sympathy of friends will not heal the wound of the bereaved parents, but it may soothe some of the pain to know that many sincerely condole with them.

---

In a case of profound , a . may arrive when the ? may well be made regarding the necessity of emptying the : Unless the conditions be grave the physician should not be expected nor * his reputation by making an abdominal §—*Weekly Medical Review.*

Brain softening will at some time put a . to that ¶r.

---

The Medical Age reports that his Satanic Majesty, while visiting earth for a cargo of sulphur to be used in his dominions, was shown a sample of iodoform. He immediately countermanded the sulphur order and substituted iodoform.

Arthur S. Underwood, M. R. C. S., L. D. S., editor of the *Journal of the British Dental Association* has been appointed dental surgeon to the Dental Hospital of London.

